# Live-cell imaging of actin dynamics reveals mechanisms of stereocilia length regulation in the inner ear

**DOI:** 10.1038/ncomms7873

**Published:** 2015-04-21

**Authors:** Meghan C. Drummond, Melanie Barzik, Jonathan E. Bird, Duan-Sun Zhang, Claude P. Lechene, David P. Corey, Lisa L. Cunningham, Thomas B. Friedman

**Affiliations:** 1 Laboratory of Molecular Genetics, Section on Human Genetics, National Institute on Deafness and Other Communication Disorders, National Institutes of Health, Bethesda, Maryland 20892, USA; 2 Department of Neurobiology, Harvard Medical School and Howard Hughes Medical Institute, Boston, Massachusetts 02115, USA; 3 National Resource for Imaging Mass Spectrometry, Brigham and Women's Hospital and Harvard Medical School, Cambridge, Massachusetts 02139, USA; 4 Division of Genetics, Department of Medicine, Brigham and Women's Hospital and Harvard Medical School, Cambridge, Massachusetts 02139, USA; 5 Section on Sensory Cell Biology, National Institute on Deafness and Other Communication Disorders, National Institutes of Health, Bethesda, Maryland 20892, USA

## Abstract

The maintenance of sensory hair cell stereocilia is critical for lifelong hearing; however, mechanisms of structural homeostasis remain poorly understood. Conflicting models propose that stereocilia F-actin cores are either continually renewed every 24–48 h via a treadmill or are stable, exceptionally long-lived structures. Here to distinguish between these models, we perform an unbiased survey of stereocilia actin dynamics in more than 500 utricle hair cells. Live-imaging EGFP-β-actin or dendra2-β-actin reveal stable F-actin cores with turnover and elongation restricted to stereocilia tips. Fixed-cell microscopy of wild-type and mutant β-actin demonstrates that incorporation of actin monomers into filaments is required for localization to stereocilia tips. Multi-isotope imaging mass spectrometry and live imaging of single differentiating hair cells capture stereociliogenesis and explain uniform incorporation of ^15^N-labelled protein and EGFP-β-actin into nascent stereocilia. Collectively, our analyses support a model in which stereocilia actin cores are stable structures that incorporate new F-actin only at the distal tips.

## Abstract (web summary)

Precise control of stereocilia length by auditory hair cells is vital for normal hearing. Drummond *et al*. follow in real-time the incorporation of actin into these structures and show that while the actin core is remarkably stable, and actin polymerization is limited to their distal tips.

## Graphical abstract

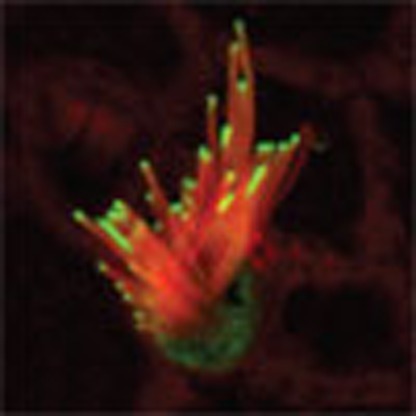

Hair cells of the inner ear transduce sound energy and head movement into afferent nerve signals that are transmitted to the brain. Hair cells owe their name to the staircase-shaped bundle of mechanosensory stereocilia (Fig. 1), which are actin-based structures that project from the apical surface into the potassium-rich endolymph of the cochlear duct and the vestibular labyrinth. These mechanosensitive cells are terminally differentiated in mammals and are not regenerated when they die^4^,^5^.

The stereocilia bundle develops from a patch of microvilli on the apical surface of a differentiating hair cell. Each microvillus undergoes a complex process of programmed elongation and thickening by increasing the length and number of polarized parallel actin filaments (F-actin) that form the paracrystalline core of each stereocilium^1^,^2^,^6^. The barbed (plus) ends of the actin filaments within each core terminate near the distal tip, which is presumed to be the site of monomer (G-actin) addition during stereocilia development^3^. Conversely, the pointed (minus) ends of the filaments terminate near the apical surface of the hair cell or perhaps extend into the rootlets that anchor stereocilia in the cuticular plate^3^,^7^,^8^. Several actin crosslinking proteins including espin, plastin/fimbrin, fascin-2 and TRIOBP contribute to the rigidity of the F-actin cores in stereocilia^8^,^9^,^10^,^11^,^12^. The staircase architecture (Fig. 1) and stiffness of the stereocilia bundle are crucial for the exquisite sensitivity of hair cells to mechanical displacements.

In contrast to the dynamic nature of microvilli, which are continuously created and disassembled with a half-life of a few minutes^13^, mammalian stereocilia are proposed to last for the lifetime of a hair cell. Evidence from transgenic mice suggests that once irreparably damaged, individual stereocilia are resorbed by the hair cell and not replaced^14^,^15^. It follows that the stereocilia actin cores of post-mitotic hair cells must be precisely maintained throughout the life of the organism. However, the molecular mechanisms that preserve these crucial mechanosensitive organelles are largely uncharacterized.

One intriguing model to explain the longevity of stereocilia is that their F-actin cores are continuously renewed via a perpetual actin treadmill. This model requires that actin monomer removal (depolymerization) at the base occurs at exactly the same rate as actin monomer addition (polymerization) at the distal tip in order to strictly maintain the steady-state length of each stereocilium. In this rapid turnover (treadmill) model, complete turnover of the F-actin core occurs every 24–48 h^16^,^17^,^18^. Actin treadmills are well studied *in vitro*^19^,^20^, and in microvilli, lamellipodia and dendritic spines *in vivo*^21^,^22^,^23^. Although actin turnover in stereocilia has been mathematically modelled^24^, direct experimental measures of polymerization and depolymerization rates have not been reported. Retrograde flow of newly incorporated and recycled G-actin in stereocilia cores was inferred from individual static images of paraformaldehyde-fixed neonatal hair cells at various time points following viral or biolistic gene gun transfection (propulsion of gold particles coated with plasmid DNA) expressing either enhanced green fluorescent protein (EGFP)-β-actin or EGFP-espin^16^,^17^,^18^.

In contrast to the rapid turnover model, the interpretation of data from other experiments indicates that stereocilia cores undergo slow protein turnover, on the order of weeks to months; a concept consistent with stereocilia cores containing exceptionally long-lived proteins (ELLPs)^25^. This model is supported by experiments in adult mice using multi-isotope imaging mass spectrometry (MIMS, measuring ^15^N-leucine incorporation) and conditional mouse mutants in which *Actb*, encoding β-actin, expression is controlled by tamoxifen-inducible *cre* recombinase^26^. These data demonstrated a ‘hotspot' of protein turnover that was restricted exclusively to the distal tip compartment in stereocilia^26^. Similar to the experimental evidence interpreted as supporting a rapid turnover model, the slow turnover model was inferred from a collection of individual static images of fixed hair cells from different animals at various time points.

In this report, we use continuous live-cell imaging in single hair cells to resolve these conflicting models. In addition, we provide MIMS data and high-resolution confocal images of over 400 stereocilia bundles from fixed hair cells transfected with EGFP-β-actin or with mutant forms of EGFP-β-actin that are unable to polymerize into filaments. Our data show that only actin in the distal tips of stereocilia is rapidly renewed and polymerization dependent, while stereocilia cores are stable structures.

## Results

### Visualization of EGFP-β-actin in living hair cells

**Figure 1:**
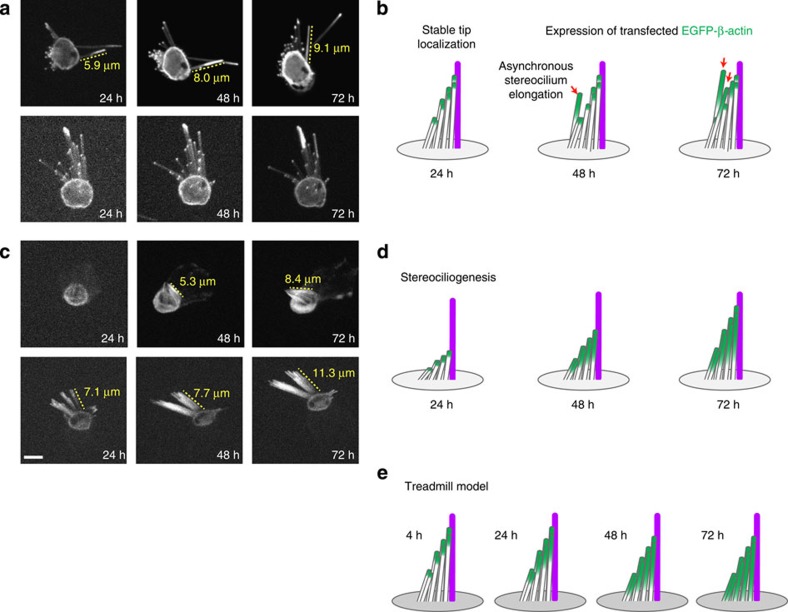
Live-cell imaging reveals different classes of EGFP-β-actin dynamics in hair cell stereocilia. (**a**) Still frames and (**b**) schematic representations of stereocilia bundles demonstrating steady-state tip localization of EGFP-β-actin (green) and asynchronous elongation (red arrow). The majority of stereocilia in the bundle retained stable tip localization of EGFP-β-actin throughout the live-imaging experiment. The lengths of stereocilia with asynchronous elongation (**a**) (yellow dashed line) were measured and are shown in yellow at each time point. (**c**) Stereociliogenesis of nascent stereocilia bundles on developing hair cells is shown in still frames and (**d**) illustrated in a schematic. Over 72 h of live imaging, stereocilia lengthen (yellow dashed lines). (**e**) Schematic representing the stereociliar F-actin treadmill hypothesis^1^,^2^,^3^. All length changes were measured in 3D using Volocity. Kinocilia are illustrated in purple. All 112 movies are available in Supplementary Movies 1, 2, 3, 4, 5, 6. Scale bar, 5 μm.

We hypothesized that live-cell imaging of hair cells biolistically transfected (that is, by gene gun) with EGFP-β-actin would enable us to collect longitudinal data from individual cells and would therefore reveal actin dynamics that cannot be observed using fixed samples. Given that utricular hair cells have longer stereocilia bundles, a higher transfection efficiency by gene gun, and were used for previous studies^16^,^17^,^18^,^26^ we chose to image utricle explant cultures. In three independent experiments, spinning-disk confocal microscopy was used to record actin dynamics in 14 transfected hair cells that displayed EGFP-β-actin localization at the tips of stereocilia (Supplementary Movie 1). We reasoned that cells exhibiting tip localization of EGFP-β-actin would offer the maximum potential to observe rapid turnover of the F-actin cores postulated to occur in the treadmill model. In the majority of stereocilia after a minimum of 62 h of continuous live imaging, we did not detect movement of EGFP-β-actin from the tips towards the bases, as would be expected if a treadmill mechanism existed. Occasionally, a single stereocilium within a bundle displayed activity consistent with an F-actin treadmill; however, these unusual stereocilia were rare (Supplementary Movie 1). Instead, we consistently captured two distinct phenomena: (1) steady-state localization of EGFP-β-actin in the distal stereocilia tips; and (2) asynchronous elongation of stereocilia (Fig. 1). In these cells, steady-state localization of EGFP-β-actin at stereocilia tips continued for up to 48 h. Beyond 48 h, elongation of some stereocilia within the bundle was recorded (Fig. 1a and Supplementary Movie 1).

**Figure 2:**
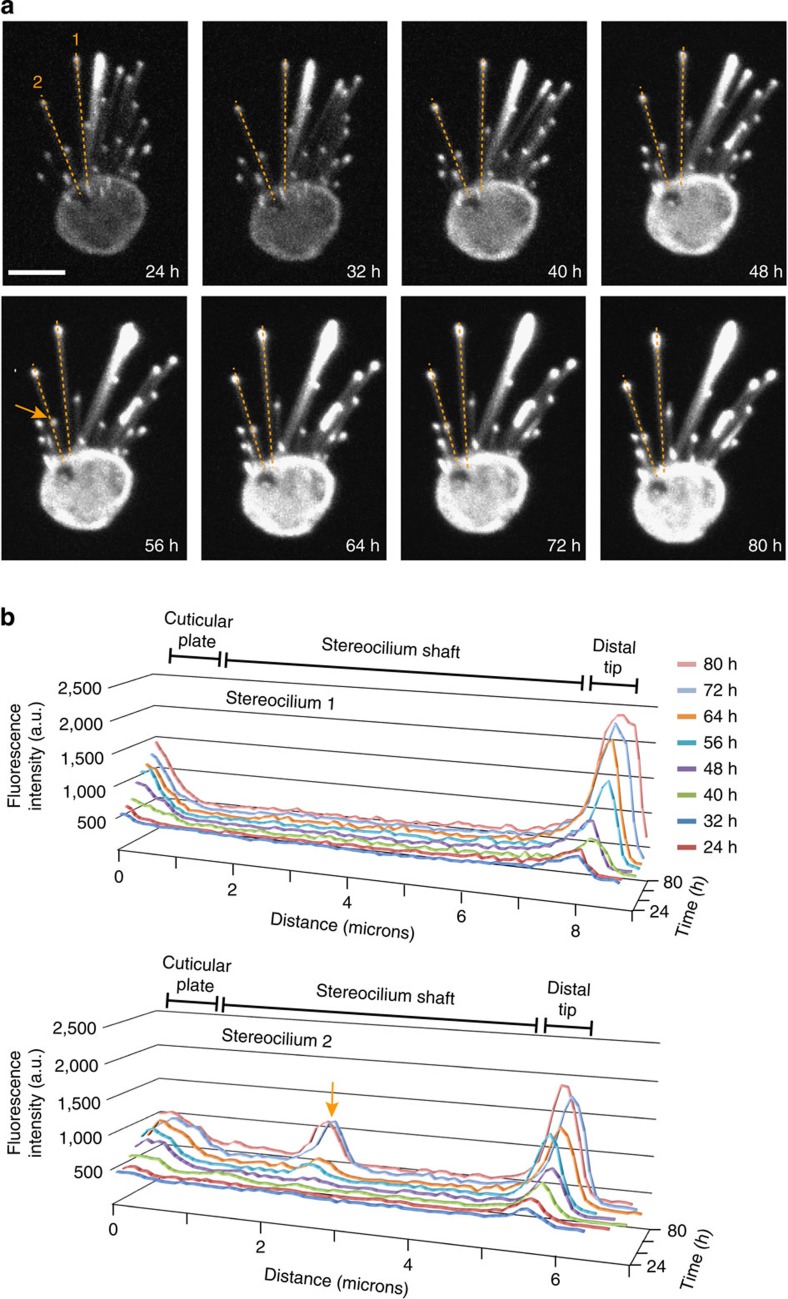
Quantification of intensity profiles along the lengths of stereocilia. (**a**) Still frames from a live-imaged hair cell with stable localization of EGFP-β-actin in the distal tip compartment. Dashed lines (orange) indicate stereocilia that were line-traced every 8 h beginning 24 h post transfection with pEGFP-β-actin. Images without line traces are available in Supplementary Fig. 1. (**b**) Line traces from stereocilia 1 and 2 demonstrate increasing levels of EGFP-β-actin in the cuticular plate and distal tip compartment, but not along the stereocilia shaft and confirm steady-state tip localization. Over the course of the experiment, additional stereocilia move in close proximity of stereocilium 2 (arrows) (**a**), resulting in extra peaks in the line traces that correspond to the distal tip of the second stereocilium. Scale bar, 5 μm.

Expression of EGFP-β-actin during our live-cell imaging experiments was under the control of a cytomegalovirus (CMV) promoter. We considered the possibility that overexpression of EGFP-β-actin resulted in a constant increase in fluorescence intensity at stereocilia tips, causing a corresponding increase in dynamic range that might mask lower levels of incorporation within the F-actin core of the stereocilium shaft. If newly synthesized EGFP-β-actin is integrated into the cores of stereocilia in a manner consistent with a treadmill, then one would expect to observe a declining gradient of EGFP fluorescence from the tips towards the bases of stereocilia. To address this possibility, we performed line scans of stereocilia from three-dimensional (3D)-volume reconstructed bundles (Fig. 2; Supplementary Fig. 1). The fluorescence intensity in the cuticular plate and distal tip compartment increased over time; however, we did not detect a corresponding increase or gradient of EGFP-β-actin along the length of the stereocilium shaft. We did detect a marginal, but uniform increase in fluorescence intensity along the length of the stereocilium that may represent a steady-state level of actin peripheral to the F-actin core (Fig. 2b). These data suggest that any incorporation of EGFP-β-actin into the F-actin core from the tips consistent with an F-actin treadmill is below the limits of detection.

### Live imaging captures stereociliogenesis

**Figure 3:**
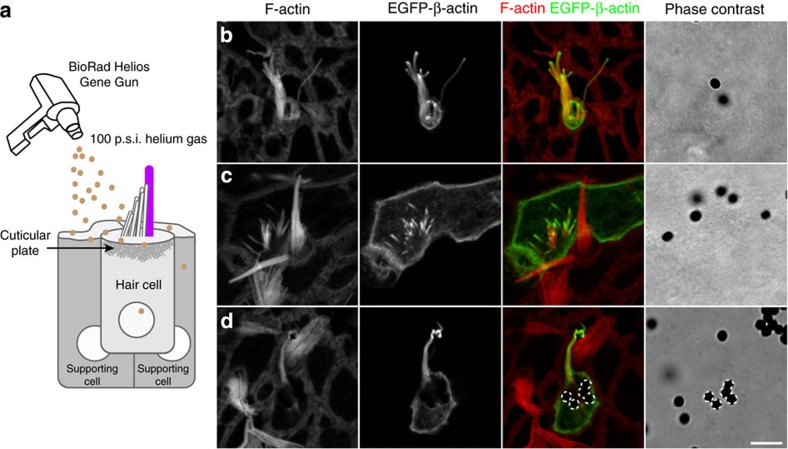
Biolistic gene gun transfection may result in damage to hair cells. (**a**) Penetration of the 1 μm gold DNA-coated particles through the cell membrane and cuticular plate of hair cells results in successful transfection of plasmid DNA; however, bullets can strike and damage stereocilia bundles in the process. (**b**–**d**) Representative images of stereocilia bundles classified as damaged and excluded from further analyses. Cells were biolistically transfected with EGFP-β-actin (green), fixed and counterstained with rhodamine phalloidin (red) to visualize all F-actin. Phase contrast shows positions of gold bullets. Dotted lines indicate gold particles on the surface of a hair cell. Scale bar, 5 μm.

While imaging the mouse utricular hair cells described above, we also captured *de novo* formation of stereocilia on the apical surfaces of seven EGFP-β-actin-expressing, newly differentiating hair cells. As the time-lapse experiment proceeded, the nascent stereocilia on these seven cells matured from microvilli and fluoresced green uniformly along their entire length consistent with the expression of EGFP-β-actin from the initiation of bundle formation (Fig. 1b and Supplementary Movies 2 and 3). To examine the extent to which stereociliogenesis occurs in the neonatal utricle, we performed a second set of three independent experiments in which we imaged every EGFP-β-actin-transfected cell in each utricle culture. The plasmid-coated gold particles used in gene gun transfection are 1 μm in diameter and are expected to periodically strike and damage stereocilia bundles (Fig. 3a). Therefore, hair cells in which stereocilia were fused, bent or missing (39/112) were excluded from the analyses (Fig. 3b–d, Table 1, Supplementary Movies 4, 5, 6). While actin dynamics at the tips of stereocilia may be influenced by a functional MET channel^27^, the F-actin core is not known to be directly affected by the presence of tip links, and therefore splayed bundles were included in the analyses. We found that stereocilia bundles in these additional 73 EGFP-β-actin-transfected hair cells all demonstrated one of the following two types of actin dynamics: steady-state tip localization with or without asynchronous elongation, or stereociliogenesis. Therefore, out of a total of 73 cells over six independent experiments, EGFP-β-actin remained steady state at the tips of morphologically stable stereocilia with or without asynchronous elongation in 53% of cells (29/73). Stereociliogenesis was observed in 60% of cells (44/73; Table 1), consistent with data indicating that 50% of mouse utricular hair cells differentiate and develop hair bundles during the first two weeks of postnatal development^28^,^29^. All of these live-imaging movies are available for viewing (Supplementary Movies 1, 2, 3, 4, 5, 6).

### EGFP-β-actin in stereocilia of fixed hair cells

**Figure 4:**
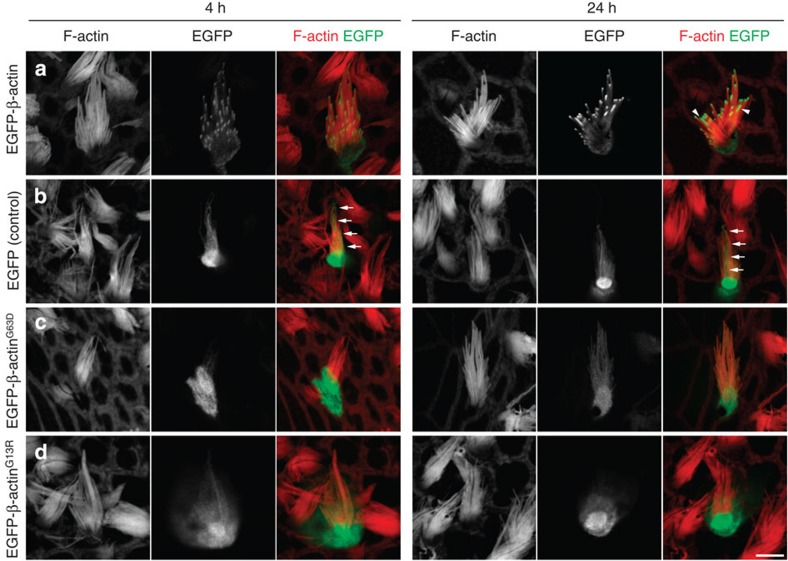
Localization of EGFP-β-actin at stereocilia tips is polymerization dependent. Localization of wild-type EGFP-β-actin, EGFP alone (negative control), and mutant EGFP-β-actin^G63D^ or mutant EGFP-β-actin^G13R^ (green) at 4 and 24 h post transfection. (**a**) Wild-type EGFP-β-actin was enriched at stereocilia tips with diffuse labelling along the shafts at 4 and 24 h post transfection. Consistent with our live-cell imaging observations, two stereocilia appear to have elongated from the distal end (arrowheads). In contrast, (**b**–**d**) EGFP-β-actin^G63D^, EGFP-β-actin^G13R^ and the EFGP control were present diffusely throughout the hair cell body, stereocilia bundle and occasionally the kinocilium (arrows) at 4 and 24 h. F-actin is labelled with rhodamine phalloidin (red). Scale bar, 5 μm.

Given that our data obtained from live-cell imaging are in direct opposition to previously published static, fixed-cell images^16^,^17^,^18^, we also performed fixed-cell confocal imaging of EGFP-β-actin-expressing hair cells. While live-cell imaging has the advantage of obtaining longitudinal data from individual cells, laser excitation intensity must be delicately balanced to limit EGFP-photobleaching and the generation of radical oxygen species. Confocal microscopy of fixed cells allows for the use of greater laser excitation intensity and a phalloidin counterstain to visualize the F-actin cores of stereocilia, and can yield images with higher resolution because the mounting media is optically matched to the objective lens. Similar to data we obtained with live-cell imaging, 49% (50/103) of biolistically transfected stereocilia bundles showed evidence of damage and were excluded from further analysis. We observed tip localization of EGFP-β-actin in 100% (14/14) of undamaged transfected stereocilia bundles 4 h post transfection (Fig. 4a, Table 1), consistent with previously published data^16^,^17^,^18^. However, in contrast to previous reports, we found that cells expressing EGFP-β-actin imaged 24 h post transfection displayed variability in the localization of the fluorescent signal. Specifically, 55% of morphologically intact transfected cells (22/40) still had EGFP-β-actin enriched at the tips of the stereocilia bundle, with weak uniform labelling of stereocilia shafts, whereas the remaining 45% (18/40) of bundles had uniformly strong labelling of stereocilia (Fig. 4a, Table 1). These data are consistent with our live-imaging data and support our conclusions that EGFP-β-actin dynamics in hair cell stereocilia fall into two distinct categories: (1) enrichment at tips with or without asynchronous elongation; and (2) fully labelled stereocilia bundles.

### Actin polymerization is necessary for tip localization

To investigate whether weak uniform EGFP-β-actin labelling along the lengths of stereocilia could be explained by diffusion from the cell body into the stereocilia bundle, we transfected neonatal utricle cultures with EGFP-β-actin mutants (either p.G13R or p.G63D) that fold correctly but are unable to incorporate into filaments^30^,^31^. We reasoned that EGFP-β-actin produced in the cell body might diffuse freely into the space surrounding the paracrystalline actin core and into the tip compartment without incorporating into the F-actin core. We transfected hair cells with either wild-type β-actin, mutant β-actin or an EGFP-only negative control and imaged hair cells fixed at 4 and 24 h post transfection. Unlike wild-type EGFP-β-actin, we did not observe accumulation of mutant EGFP-β-actin^G13R^ or EGFP-β-actin^G63D^ at stereocilia tips in the vast majority (124/130) of fixed hair cells at 4 and 24 h post transfection (Fig. 4c,d, Table 1). However, EGFP-β-actin^G13R^ or EGFP-β-actin^G63D^ was present in the stereocilia shaft at a similar level observed for wild-type EGFP-β-actin. In 7% (6/90) of hair cells transfected with EGFP-β-actin^G63D^, we did detect enrichment of EGFP fluorescence at the tips of stereocilia that we did not observe in EGFP-β-actin^G13R^-transfected hair cells. These data are consistent with published biochemical data demonstrating that EGFP-β-actin^G13R^ is incapable of polymerizing into filaments in the presence of wild-type β-actin^31^, while β-actin^G63D^ can form small filaments in the presence of a high concentration of wild-type β-actin^30^. These data demonstrate that incorporation of actin monomers into filaments is required for trafficking of EGFP-β-actin to the tips of hair cell stereocilia, retention of EGFP-β-actin at the tips or both. Control experiments in which hair cells were transfected with EGFP only (that is, no actin) demonstrated that complete fluorescent labelling of stereocilia shafts can be achieved solely by diffusion of proteins from the cell body (Fig. 4b, Table 1). Thus, our data indicate that some macromolecules in the cell body are not restricted from diffusion into the stereocilia bundle compartment.

### Photoconversion of dendra2-β-actin at stereocilia tips

To further investigate the dynamics of β-actin at the tips of stereocilia, we biolistically transfected a plasmid encoding dendra2-β-actin into cultured neonatal utricles. Dendra2 is a monomeric green fluorescent molecule that can be irreversibly converted to emit red fluorescence by exposure to 405-nm (and to a lesser extent 488 nm) light. The red variant of the molecule is photo-stable, making it possible to image a population of photoconverted actin molecules for several hours^32^. We used photoconversion to track the movement of a specific population of differentially labelled fluorescent actin.

**Figure 5:**
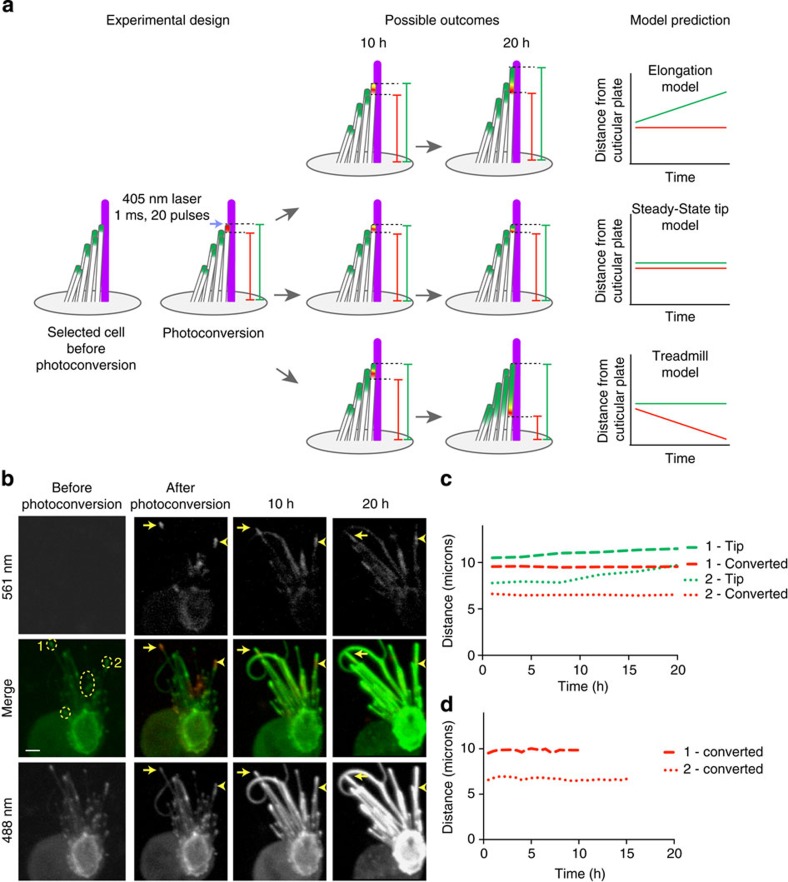
Photoconversion of dendra2-β-actin reveals stable actin in stereocilia cores. (**a**) Schematic of the experimental design, possible outcomes of the experiment and theoretical data of each predicted model. Cultured utricles biolistically transfected with pDendra2-β-actin were mounted onto a spinning-disk microscope for live imaging. The distal tip compartments of stereocilia were targeted with a 405 nm laser to photoconvert dendra2 from green to red. Dashed lines indicate the tip of the stereocilium and the lower boundary of the photoconverted region. (**b**) Time-lapse images from a representative cell before photoconversion, immediately after photoconversion and at 10 and 20 h post-photoconversion. Photoconverted regions of interest are denoted by a yellow dashed circles. A 488 nm laser and 561 nm laser were used to excite non-photoconverted dendra2-β-actin (green) and photoconverted dendra2-β-actin (red), respectively. During photoconversion, dendra2-β-actin in the cuticular plate was also converted and can be seen moving into the shafts of stereocilia (Supplementary Movie 7). (**c**) The distance from the boundary of the photoconverted dendra2-β-actin (red) and the distance from the distal tip of the stereocilium (green) were measured every 8 h and plotted for stereocilia tips 1 and 2 indicated by the yellow arrow and arrowheads, respectively, in panel **b**. Measurements were made with both the green and red channels displayed to determine the insertion point of the photoconverted stereocilium into the cuticular plate. The distance from the lower boundary of the photoconverted dendra2-β-actin to point of insertion of the stereocilium into the apical surface of the hair cell does not change, consistent with steady-state tip localization of photoconverted dendra2-β-actin. In contrast, the distance from the distal tip to the apical surface increases over time, consistent with stereocilia elongation. (**d**) Automated measurements were made using Volocity software to plot the distance from the centroid of the photoconverted dendra2-β-actin to the edge of the non-photoconverted cuticular plate (green). Scale bar, 5 μm.

Locations within dendra2-β-actin-expressing hair cells were specifically targeted with a 405 nm laser to photoconvert dendra2-β-actin from green (dendra2-β-actin^GREEN^) to red (dendra2-β-actin^RED^). Optical sections through the entire hair cell were taken every 30 min for 24 h. The dynamics of photoconverted dendra2-β-actin^RED^ were analysed using two independent methods (Fig. 5). We used Volocity 3D image analysis software to generate volume-rendered 3D images. Measurements were made at 6-h intervals to determine (1) the distance from the tip of a stereocilium to its insertion point in the hair cell cuticular plate and (2) the distance from the lower boundary of the photoconverted population of actin to the insertion point in the cuticular plate (Fig. 5). Using these methods, we found that the position of the photoconverted population of dendra2-β-actin^RED^ remained stable relative to the stereocilium insertion point, while the total length of the stereocilium increased through addition of non-photoconverted dendra2-β-actin ^GREEN^ distal to the photoconverted dendra2-β-actin^RED^ (Fig. 5, Supplementary Movie 7). To avoid any observer bias in these direct measurements, we also utilized Volocity to generate an automated method of defining and quantifying the distance from the photoconverted dendra2-β-actin^RED^ to the edge of the cuticular plate, which fluoresced green with non-photoconverted dendra2-β-actin^GREEN^ (Fig. 5d). In agreement with our direct measurements, the software-generated automated measurements indicate that the position of the photoconverted dendra2-β-actin^RED^ remains stable until the point at which the signal is below the limits of detection (usually after about 16 h of imaging). These data are consistent with our observations using live imaging of EGFP-β-actin demonstrating that stereocilia can elongate their F-actin cores, but do not treadmill.

### Developing hair bundles are detected by MIMS

**Figure 6:**
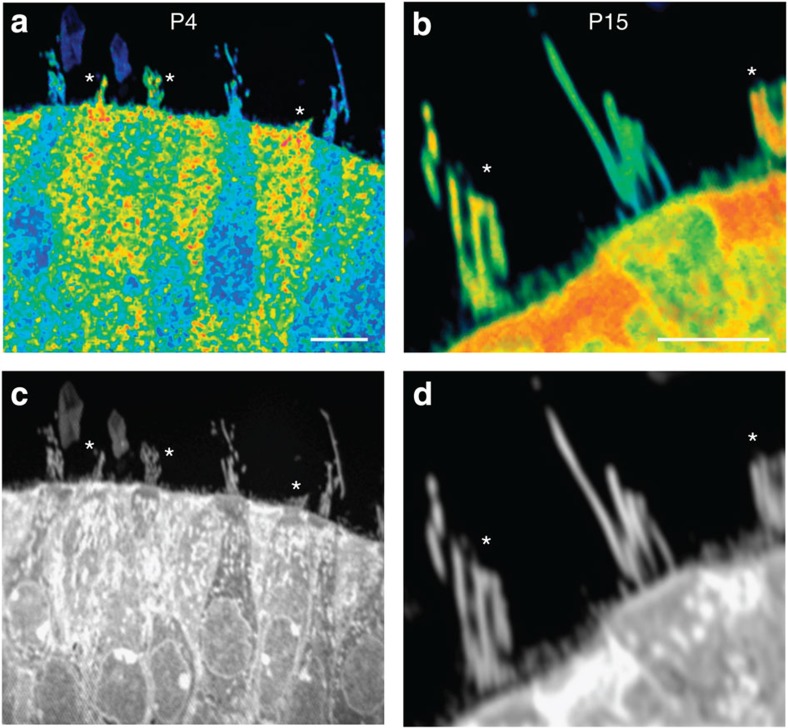
Postnatal stereociliogenesis is detected with MIMS. Representative images of mouse utricles at postnatal day 4 (P4) and P15. The ^15^N/^14^N ratio is illustrated in pseudocolour, with blue indicating low and red indicating high incorporation of ^15^N into newly synthesized proteins. (**a**) At P4, three of the six stereocilia bundles in the field are small and show high levels of protein incorporation (asterisks); they are consistent with being immature bundles that developed after ^15^N feeding began at P0. (**b**) At P15, bundles are approaching their mature sizes, but one has little incorporation of new protein (centre), whereas two show high incorporation of new protein (asterisks) consistent with development after P0. (**c**,**d**) Images of the same fields, indicating total protein. Scale bar, 5 μm.

In addition to live imaging, a second method was used to capture stereociliogenesis in postnatal utricles *in vitro*. We examined the incorporation of newly synthesized protein into growing stereocilia using MIMS^26^, a method that reveals the presence of selected isotopes in thin sections of tissue. To label newly synthesized protein with ^15^N, a rare but stable isotope of nitrogen, we transferred newborn mouse pups to foster mothers whose food contained ^15^N-leucine. Pups continued to nurse from these ^15^N foster mothers for 4 or 15 days before sacrifice, so that protein synthesized after birth would be ^15^N-labelled. We used MIMS to reveal the localization of both postnatally synthesized protein containing ^15^N and prenatally synthesized protein containing ^14^N, as previously described^26^. The location of postnatally synthesized protein was indicated as the ratio of ^15^N/^14^N at every point in the image (Fig. 6). At postnatal day 4, most hair bundles contained relatively low levels of ^15^N, suggesting that they had developed before birth. However, about a quarter of stereocilia bundles (8 out of 34 observed with MIMS) were shorter in length and had higher ^15^N levels, suggesting that they had developed postnatally. At postnatal day 15 most bundles had reached a mature length, but nearly half (20/43) showed high ^15^N incorporation, indicating postnatal development. Thus utricular stereocilia can be labelled along their entire length with labelled protein if they develop after birth. These MIMS data, together with our observations using both EGFP-β-actin and photoconvertible dendra2-β-actin, are consistent with a model in which stereocilia F-actin cores, once formed, do not treadmill. Instead, stereocilia are exceptionally long-lived structures with newly synthesized F-actin restricted to the distal tips.

## Discussion

The rapid and slow turnover models for the maintenance of hair cell stereociliar F-actin cores are seemingly irreconcilable^16^,^17^,^18^,^26^. Our data from live imaging of hair cells and static images from fixed EGFP-β-actin-transfected tissue and MIMS show that (a) turnover of F-actin is restricted to the distal stereocilia tip compartment, (b) the accumulation of actin at stereocilia tips requires actin polymerization, and (c) stereociliogenesis, during which nascent bundles elongate and thicken their F-actin cores, results in stereocilia cores labelled along their length with EGFP-β-actin.

The use of live-cell imaging allowed us to longitudinally observe actin dynamics in the same stereocilia bundle and resolve several long-standing questions. Had we only observed images of different fixed samples at multiple post-transfection intervals, the rapid turnover stereocilia treadmill model would have been a plausible explanation for our data, given the well-documented role of actin treadmilling in other types of cellular protrusions, such as dendritic spines^21^,^22^. In fact, when we performed similar fixed-cell experiments (Fig. 4), two patterns of EGFP-β-actin localizations within stereocilia bundles at 4 and 24 h post transfection were observed, further underscoring the importance of following EGFP-β-actin dynamics in the same cell over time by live imaging. As a specific example, it is impossible to distinguish between elongation from the tips of stereocilia and a retrograde actin treadmill unless multiple time points are collected for the same hair cell bundle and 3D measurements are made.

A few caveats need to be considered when interpreting our data. First, caution must be exercised when assigning meaning to the percentage of cells that show a particular EGFP-β-actin localization. Of the 539 cells that we imaged, 217 either died shortly after the initiation of the experiment or their stereocilia bundles were extensively damaged by the mechanical trauma of biolistic (gene gun) transfection. Successful biolistic transfection requires that the plasmid-coated 1 μm gold particles penetrate the cellular membrane without striking the prominent but fragile stereocilia bundle^33^. Thus, it is not surprising that a significant proportion of cells transfected with this technique are damaged. Moreover, the proportion of cells categorized as ‘enriched at tips' versus ‘fully labelled bundle' may reflect increased efficiency of transfecting a nascent hair cell compared with that of a mature hair cell. In mature hair cells, the dense, heavily crosslinked F-actin comprising the cuticular plate at the apical surface (Fig. 3a) is likely to hinder biolistic transfection, whereas the gold particle may more easily penetrate the developing hair cell through an immature cuticular plate. Finally, overexpression of EGFP-β-actin may itself damage a hair cell, as actin concentrations in cells are closely controlled^34^. In particular, it is unclear whether the observed asynchronous elongation of an individual stereocilium in a bundle is a normal occurrence, a response to damage from biolistic transfection or an artifact of overexpressing EGFP-β-actin in cultured hair cells. Intravital imaging of hair cells in live animals will be necessary to resolve these open questions.

Data obtained using non-polymerizable missense mutants EGFP-β-actin^G13R^ or EGFP-β-actin^G63D^ indicate that the actin population in the distal tip compartment of stereocilia is likely composed of F-actin. These data, along with evidence from a companion paper^35^ that the tip compartment is renewed at a faster rate than the actin core, raise questions about the role(s) of this dynamic F-actin pool in this distinct subcellular compartment. In addition to its established functions in cell structure and motility^36^,^37^, F-actin also serves as a scaffold or barrier to restrict the trafficking of vesicles or molecules^38^,^39^. F-actin in the distal tip compartment of stereocilia may be an extension of the paracrystalline core or perhaps this distinct population of actin has an alternative role in regulating stereocilia tip dynamics in close proximity to the site of mechanoelectric transduction^27^,^40^.

A model of stereocilia dynamics in which a zone of rapid turnover is immediately adjacent to a long-lived F-actin core is not without precedence in other eukaryotic structures. ELLPs that elude turnover^25^ are found in the nuclear pore complexes of non-dividing cells. Some nucleoporins have half-lives on the order of months to years, while structurally adjacent nucleoporins encoded by different genes are rapidly replaced^25^. Emerging evidence supports the idea that a protein's half-life is not necessarily uniform throughout the cell or even within the same subcellular compartment. Instead, a protein's stability likely depends on several factors, including post-translational modifications, interacting partners and subcellular localization^41^,^42^. Our data are consistent with the view that actin and actin-binding proteins comprising stereocilia cores are ELLPs, though the modifications that allow this long-term persistence remain elusive.

The repair mechanisms of long-lived structures such as stereocilia cores remain to be discovered. For example, gaps in phalloidin-stained F-actin cytoskeletons of stereocilia are particularly abundant after noise trauma or in the absence of either β- or γ-actin^14^,^15^. Toyama and Hetzer^41^ have suggested that unrepaired damaged ELLPs may contribute to aging processes. Furthermore, some of the homeostatic mechanisms that maintain hair cells and preserve stereocilia function include the secretion of protective molecules from supporting cells and active remodelling of protein complexes that comprise tip links^43^,^44^,^45^. A fuller understanding of homeostasis and repair mechanisms within each of the various stereocilia compartments during aging may have translational potential for preservation and restoration of healthy hearing.

## Methods

### Plasmids and biolistic transfection

For live-cell imaging experiments, human *ACTB* cDNA (NM_001101.3, identical amino acid sequence to mouse β-actin) cloned into the Green FP expression vector (BD Biosciences, San Jose, CA) was used to prepare gold particles for biolistic transfection as previously described^42^ with minor modifications. Specifically, we used antibiotics in the culture media (see Organ culture and live imaging below) and reduced the BioRad Helios Gene Gun firing pressure to 100 p.s.i. in order to reduce damage caused by biolistic transfection. The p.G13R and p.G63D substitutions of β-actin were introduced into pEGFP-β-actin using site-directed mutagenesis (Stratagene, La Jolla, CA) with primers 5′-CGTCGTCGACAAC**C**GCTCCGGCATGTG-3′, 5′-CACATGCCGGAGC**G**GTTGTCGACGACG-3′, 5′-CCCAGAGCAAGAGAG**A**CATCCTCACCCTGAA-3′ and 5′-TTCAGGGTGAGGATG**T**CTCTCTTGCTCTGGG-3′, respectively (point mutation in bold). pDendra2-β-actin was cloned using InFusion (Clontech, Mountain View, CA) with primers 5′-GTGTACAAGACTCGAGCCACCATGGATGATGATATCG-3′ and 5′-TAGATCCGGTGGATCCCTAGAAGCATTTGCGGT-3′ to amplify the cDNA of *ACTB* and insert it between the XhoI and BamHI sites of pDendra2-C (Clontech, Mountain View, CA). The DNA sequences of all plasmid inserts were verified by Sanger sequencing.

### Organ culture and live imaging

C57Bl6/J pups were killed by decapitation at postnatal day 2–5 in accordance with National Institutes of Health Institutional Animal Care and Use Committee-approved guidelines under animal study protocol #1263. Utricles were dissected in L-15 media (Life Technologies, Carlsbad, CA, USA) and cultured on 2 mg ml^−1^ collagen matrices in DMEM/F-12 with 20 mM L-glutamine, 7% fetal bovine serum, and 1 unit per ml penicillin-G. One hour before biolistic transfection, growth media was replaced with antibiotic-free media. Transfections were done using a Helios gene gun and endotoxin-free plasmid DNA (NucleoBond Xtra EF, Macharey-Nagel). Twenty-four hours post transfection, cultures were transferred to phenol-red free media supplemented with penicillin-G and mounted under a platinum and nylon harp^46^ at 37 °C in a humidified 6% CO_2_ atmosphere with the stereocilia facing the coverglass and objective.

Cells transfected with pEGFP-β-actin were live-imaged on a Zeiss Cell Observer spinning disc microscope (× 63 plan-apochromat, NA 1.4) for 62–92 h. Z-stack volumes (138 × 0.4 μm) were captured at 1-h intervals. For photoconversion experiments with pDendra2-β-actin, transfected cultured cells were imaged for 24 h and z-stack volumes were captured at 30 min intervals. Dendra2-β-actin was converted by exposing a defined region of interest to 405 nm laser light for 15 × 1-ms bursts at 6% of the total laser power (UltraView, Perkin Elmer, Akron, OH, Laser Module 2). Post-acquisition image analyses were performed using ImageJ (http://imagej.nih.gov/ij/) and Volocity (Perkin Elmer, Akron, OH, USA). For automated analysis of dendra2-β-actin experiments (Fig. 5d), the red photoconverted populations of dendra2-β-actin and the green non-photoconverted dendra2-β-actin cuticular plate were identified mathematically. The distance from the centroid of the red population to the edge of the cuticular plate was measured for each time point.

### Fixed-cell imaging

Mutant expression constructs pEGFP-β-actin^G63D^ and pEGFP-β-actin^G13R^, the wild-type EGFP-β-actin control and the EGFP-only control were biolistically transfected into cultured utricles from neonatal mice (P2–P5) and fixed in 4% paraformaldehyde at 4 and 24 h post transfection for imaging^17^,^18^,^33^. After fixation, samples were washed in phosphate-buffered saline, counterstained with rhodamine-conjugated phalloidin and mounted in ProLong Antifade Gold (Life Technologies).

### Multi-isotope imaging mass spectrometry

Mouse pups born to normal mothers were switched at birth to nurse from mothers who had been fed for a month or more with food containing ^15^N-leucine (∼1.25% relative to ^14^N-leucine). Pups were killed after 4 or 15 days. Utricles were fixed overnight at 4 °C in 4% formaldehyde+10% glutaraldehyde in cacodylate buffer, then processed for plastic embedding and thin sectioning.

We performed MIMS as described previously^47^,^48^. Briefly, an accelerated beam of Cs^+^ ions, focused to a 30-nm spot, was scanned across the surface of a thin section, sputtering molecules from the surface. Anionic molecules were accelerated back and analysed with a double sector mass spectrometer. ^15^N was detected as the cyanide ion ^12^C^15^N^−^ at mass 27 and compared with ^12^C^14^N^−^ at mass 26 for each pixel in the scanned image. Incorporation was calculated from the natural abundance of ^15^N and the relative abundance of ^15^N in the food^26^. To show incorporation as a percentage in the image, we used a hue saturation intensity transformation in which the hue represents percentage and the intensity indicates reliability.

## Author contributions

All authors participated in the conception and design of the study; M.C.D., M.B. and D.-S.Z. performed the research; all authors contributed to the analysis of data and wrote the manuscript.

## Additional information

**How to cite this article:** Drummond, M. C. *et al*. Live-cell imaging of actin dynamics reveals mechanisms of stereocilia length regulation in the inner ear. *Nat. Commun*. 6:6873 doi: 10.1038/ncomms7873 (2015).

## Supplementary Material

Supplementary Information Supplementary Figure 1

Supplementary Movie 1 Montage of all cells classified as “steady-state tip localization”. Displayed as a single movie, each region of interest is presented in a single box as a maximum intensity projection. All movies were cropped to identical size without changing magnification and the brightness and contrast was uniformly normalized to 0.3% saturation for each frame of each movie using the “Enhance contrast – normalize” function in ImageJ.

Supplementary Movie 2 Montage of cells classified as “stereociliogenesis” – group 1. Displayed as a single movie, each region of interest is presented in a single box as a maximum intensity projection. All movies were cropped to identical size without changing magnification and the brightness and contrast was uniformly normalized to 0.3% saturation for each frame of each movie using the “Enhance contrast – normalize” function in ImageJ.

Supplementary Movie 3 Montage of cells classified as “stereociliogenesis” – group 2. Displayed as a single movie, each region of interest is presented in a single box as a maximum intensity projection. All movies were cropped to identical size without changing magnification and the brightness and contrast was uniformly normalized to 0.3% saturation for each frame of each movie using the “Enhance contrast – normalize” function in ImageJ.

Supplementary Movie 4 Montage of cells classified as “damaged” – group 1. Displayed as a single movie, each region of interest is presented in a single box as a maximum intensity projection. All movies were cropped without changing magnification and the brightness and contrast was uniformly normalized to 0.3% saturation for each frame of each movie using the “Enhance contrast – normalize” function in ImageJ.

Supplementary Movie 5 Montage of all cells classified as “damaged” – group 2. Displayed as a single movie, each region of interest is presented in a single box as a maximum intensity projection. All movies were cropped without changing magnification and the brightness and contrast was uniformly normalized to 0.3% saturation for each frame of each movie using the “Enhance contrast – normalize” function in ImageJ.

Supplementary Movie 6 Montage of all cells classified as “damaged” – group 3. Displayed as a single movie, each region of interest is presented in a single box as a maximum intensity projection. All movies were cropped without changing magnification and the brightness and contrast was uniformly normalized to 0.3% saturation for each frame of each movie using the “Enhance contrast – normalize” function in ImageJ.

Supplementary Movie 7 Movie of cell shown in Figure 5B

## Figures and Tables

**Table 1:** EGFP localization in sensory hair cells.

	Time after transfection (h)	Transfected cells	Damaged cells*	Cells included in analysis	Stereocilia tips	Uniformly labelled stereocilia	Primarily cell body, weakly labelled stereocilia
EGFP-β-actin	24–96† (live imaged)	112	38 (34%)	73	29 (40%)	44 (60%)	0
	4	21	7 (33%)	14	14 (100%)	0	0
	24	82	43 (52%)	40	22 (55%)	18 (45%)	0
EGFP (control)	4	48	12 (25%)	36	0	0	36 (100%)
	24	46	17 (37%)	29	0	0	29 (100%)
EGFP-β-actin^G63D^	4	21	6 (29%)	15	1 (7%)	8 (53%)	6 (40%)
	24	143	68 (48%)	75	5 (7%)	22 (29%)	48 (48%)
EGFP-β-actin^G13R^	4	6	2 (33%)	4	0	0	4 (100%)
	24	60	24 (40%)	36	0	0	36 (100%)
Total		539	217 (40%)	322	72	92	159

EGFP, enhanced green fluorescent protein.

^*^Cells classified as damaged were not included in further analyses.

^†^96 h was the maximum duration of live imaging.
