# Supplementary material for: Live-cell imaging of actin dynamics reveals mechanisms of stereocilia length regulation in the inner ear
Source: Nat Commun. 2015 Apr 21;6:6873. doi: 10.1038/ncomms7873 (PMC4411292; doi:10.1038/ncomms7873)
Supplement: Supplementary Information — Supplementary Figure 1 [file ncomms7873-s1.pdf]

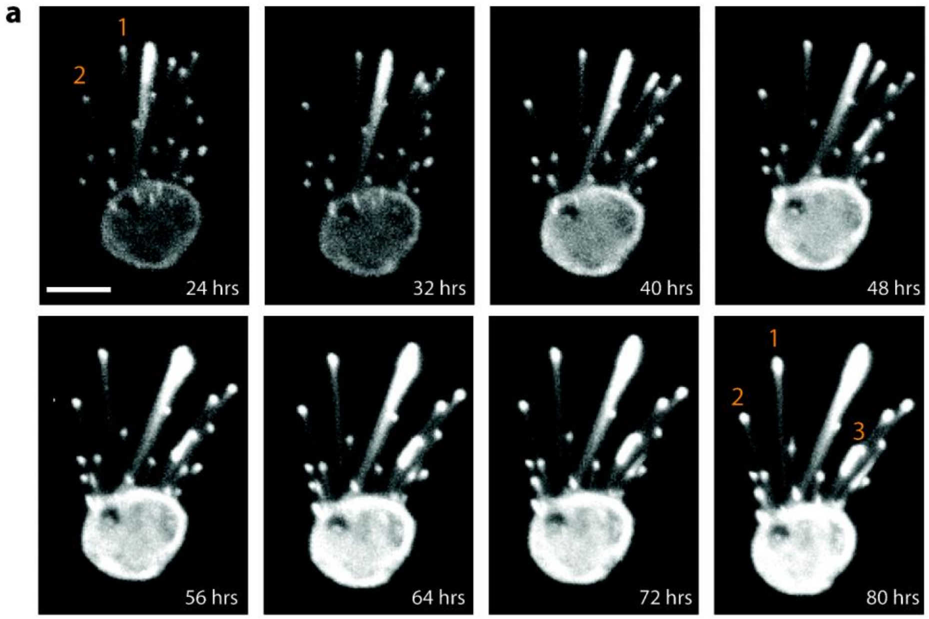

Supplementary Figure 1 | Images of stereocilia used in Figure 3 without dashed lines

Scale bar, 5 μm.
